# Large fiber contribution to symptoms in chemotherapy-induced peripheral neuropathy in cancer survivors

**DOI:** 10.1007/s00520-026-10736-5

**Published:** 2026-05-07

**Authors:** Jon D. Levine, Bruce A. Cooper, Gary Abrams, Carolyn Harris, Steven M. Paul, Yvette P. Conley, Marilyn J. Hammer, Christine Miaskowski

**Affiliations:** 1 School of Medicine, University of California, San Francisco, CA USA; 2 School of Nursing, University of California, San Francisco, CA USA; 3 School of Nursing, University of Pittsburgh, Pittsburgh, PA USA; 4 Phyllis F. Cantor Center, Dana Farber Cancer Institute, Boston, MA USA

**Keywords:** Affective touch, Balance, C-Tactile afferents, Chemotherapy-induced peripheral neuropathy, Large fiber function, Numbness, Pain, Touch sensation, Vibration

## Abstract

**Purpose:**

Chemotherapy-induced peripheral neuropathy (CIPN) is associated with a large amount of interindividual variability in signs and symptoms. The purposes were to use latent profile analysis to identify subgroups of survivors with distinct lower extremity (LE) loss-of-function CIPN profiles; evaluate for differences in demographic, clinical, and pain characteristics between the profiles and characteristics associated with membership in the more severe profile; and examine relationships between the profiles and measures of large fiber loss and C-tactile fiber function.

**Methods:**

LE loss-of-function CIPN profiles were created using measures of worst pain, loss of light touch sensation, loss of cold sensation, loss of pain sensation, vibration threshold, and two balance measures.

**Results:**

Of the 405 survivors evaluated, two distinct profiles were identified: less severe loss of LE function (76.5%) and more severe loss of LE function (23.5%). Risk factors for membership in the more severe profile included being older, male, and having lower functional status. In terms of the loss of large fiber function, survivors in the more severe class had four more sites on average that lost light touch sensation; their vibration thresholds were 1.5 times higher; and their ratings of numbness were significantly higher. For C-tactile fiber function, significant between-group differences were found in survivors’ ratings of the severity of sensitive skin and unpleasantness.

**Conclusions:**

Findings suggest that distinct “CIPN phenotypes” can be identified in cancer survivors. Detailed phenotyping and molecular characterization of various CIPN phenotypes will lead to the development and testing of targeted and personalized interventions.

## Introduction

Chemotherapy-induced peripheral neuropathy (CIPN) is the most common neurologic complication of cancer treatment [1, 2]. CIPN results in a variable set of signs and symptoms [3, 4]. Sensorimotor complaints occur predominantly in the extremities and can include pain, numbness, tingling, and burning sensations; gait and balance disturbances; difficulty with fine motor skills; reductions in vibratory sense, touch, and proprioception; and progressive loss of deep tendon reflexes. However, a large amount of interindividual variability exists in the CIPN phenotype (i.e., both in the positive and negative expressions of signs and symptoms) [4–7].

While the etiology of neuropathic pain is the most common way to classify patients (e.g., diabetic neuropathy, post-herpetic neuralgia, and CIPN), recent work used newer analytic techniques to identify subgroups of patients with distinct neuropathic pain profiles [8–13]. For example, in one study [11], self-report and objective data from patients with polyneuropathy, peripheral nerve injury, radiculopathy, and post-herpetic neuralgia were evaluated. Using cluster analysis, three subgroups of patients were identified and named based on the grouping of the subjective and objective data (i.e., “sensory loss,” “thermal hyperalgesia,” and “mechanical hyperalgesia”). Of note, each subgroup included patients with all the pain conditions that were evaluated in the study. The authors concluded that these three subgroups or “pain profiles” that were identified using cluster analysis may be related to different underlying mechanisms and that this type of classification may be useful in the selection of patients for studies of mechanistically based interventions. However, no studies have used subjective and objective CIPN characteristics to identify distinct “CIPN profiles” in cancer survivors.

An important consideration in understanding the mechanisms of CIPN is the role of small-diameter versus large-diameter sensory neurons. In the most common classification scheme, fast conducting, large myelinated Aβ fibers (i.e., large fibers) mediate nonnociceptive sensations, and thinly myelinated A-delta and small unmyelinated C-fibers (i.e., small fibers) mediate nociceptive sensations [14, 15]. In terms of CIPN, the administration of neurotoxic chemotherapy damages both small fibers and large fibers and is associated with pain and the attenuation of light touch sensation, respectively [16–19].

However, more recent evidence suggests that damage to large fibers contributes to painful sensations associated with CIPN. For example, in our previous study of survivors with CIPN [20], average pain scores were positively correlated with subjective (severity of numbness) and objective measures of large myelinated fiber function (i.e., loss of protective sensation and increases in vibration thresholds). In terms of the patients with CIPN, these findings suggest that the symptoms and objective changes in sensation from the administration of platinum- and/or taxane-containing regimens are congruent with the loss of large fiber function and associated increases in pain. Additional support for the contribution of large fibers to CIPN comes from a recent preclinical study that used a high-frequency electrical stimulus that selectively activates large myelinated afferents as a model of large fiber CIPN [21]. In this study, that evaluated the effects of duloxetine on bortezomib and paclitaxel-induced CIPN, both neurotoxic drugs produced changes that were compatible with the loss of large fiber function. Of note, duloxetine prevented the development of CIPN.

While additional research is warranted on the relative contribution of large fiber function to CIPN, another plausible hypothesis for the development of this significant clinical problem is damage to a subgroup of C fibers known as C-tactile fibers. These slow conducting unmyelinated afferents have a low temporal and spatial resolution and are thought to contribute to the perception of pleasantness during gentle stroking of hairy skin (i.e., “affective touch”) [22–24]. Evidence from preclinical models suggests that these fibers play a role in the pathophysiology of tactile allodynia [25]. Given that some patients with CIPN report tactile allodynia (e.g., burning, tender sensations from nonnoxious stimuli) [26], it is plausible that the administration of neurotoxic chemotherapy damages these fibers and results in these extremely unpleasant sensations.

Given the lack of research on interindividual variability in CIPN profiles, the purpose of this study, in a sample of survivors with CIPN (*n* = 405), was to use latent profile analysis (LPA) to identify subgroups of survivors with distinct lower extremity loss-of-function CIPN profiles (i.e., latent classes). Once the profiles were identified, differences in demographic, clinical, and pain characteristics between the profiles were evaluated and demographic and clinical characteristics associated with membership in the more severe profile were determined. In addition, relationships between the profiles and measures of large fiber loss and C-tactile fiber function were evaluated to determine their potential involvement in CIPN.

## Methods

### Survivors and settings

The current analysis is part of a larger National Cancer Institute–funded study that evaluated CIPN in cancer survivors. The methods for the larger study are described in detail elsewhere [27], and a complete description of the methods is provided in Supplemental Document 1. Of the 1450 survivors who were screened, 754 were enrolled, and 623 completed the self-report questionnaires and the study visit. Only survivors with CIPN (*n* = 405) were included in this analysis.

### Study procedures

Research nurses screened and consented the survivors over the phone; sent and asked them to complete the self-report questionnaires prior to their study visit; and scheduled the in-person assessment. At this assessment, written informed consent was obtained; questionnaires were reviewed for completeness; and objective measurements were done.

### Study measures

#### Demographic and clinical characteristics

Survivors provided information on demographic characteristics and completed the Alcohol Use Disorders Identification Test [28], the Karnofsky Performance Status (KPS) scale [29], and the Self-Administered Comorbidity Questionnaire (SCQ) [30].

#### Pain questionnaires

A detailed history of CIPN was obtained using a questionnaire from our previous [31, 32] and ongoing studies. Information was obtained on the date of onset of pain and its level of interference with function. Average and worst pain intensity over the past 24 h were assessed using a 0 (no pain) to 10 (worst pain imaginable) numeric rating scale (NRS) [33]. The 20-item PQAS was used to assess the qualities associated with CIPN [34, 35].

#### Objective measures of large fiber function

Semmes–Weinstein monofilaments (SWM; North Coast Medical, Inc., Morgan Hill, CA) were used to test light touch sensation in the lower extremities [36]. Each lower extremity was evaluated at nine locations: pad of the great, third, and fifth toes; base of the heel; dorsal surface of the metacarpophalangeal joint of the great, third, and fifth toes; midway along the tibia; and the patella. The locations were tested in random order.

SWM used for the lower extremities were 4.31, 4.56, 5.07, and 6.65. Before testing began, the patient was familiarized with the filament being used and the expected sensation was demonstrated in an area of normal sensibility. Then, with the survivor’s’ eyes closed, starting with the smallest, filaments were applied in ascending order at each site. The filaments were applied perpendicular to the skin and pressed until the filament bowed for 1.5 s. The survivor was asked to respond to the stimulus by stating the location of the light touch sensation. In contrast to Bell-Krotoski’s recommendation, two correct responses rather than one out of three at each location were considered a positive response, as a survivor could make one correct response by guessing [37]. At each location, once a positive response was identified, the next location was tested in random order. SWM measurements have good inter- and intrarater reliability and validity when calibrated and applied correctly [36, 38, 39].

Vibration threshold was tested using a biothesiometer (Bio-Medical Instrument Company; Newbury, OH). After familiarizing survivors with the sensation, vibration thresholds were tested at three sites in the lower extremities (i.e., dorsal ipsilateral joint of the great toe, medial malleolus, and patella). Following the manufacturer’s instructions, the biothesiometer was placed on the skin over the bone at each location. Beginning at zero amplitude, the amplitude of the vibration was increased until the survivor reported feeling vibration (i.e., vibration perception threshold). Then, the amplitude was turned down to zero and the procedure was repeated increasing the intensity more slowly as the initial value was approached. The survivor reported the perception of the sensation of vibration by saying, “NOW.” Each site was tested three times and a mean score was calculated. Loss of vibration thresholds can be categorized as follows: < 15 V is normal; 15 to 20 V is mild loss; 20 to 25 V is moderate loss; and > 25 V is severe loss of vibratory sensation [40].

#### Additonal measures of sensation

Cold sensation was evaluated using the Tiptherm Rod [41, 42]. Pain sensation was evaluated using the Neurotip [41]. For all of the measures of sensation, the lower extremity on the dominant side was tested.

#### Balance measures

The objective measures of balance were the Timed Get Up and Go test (TUG) [43] and the Fullerton Advanced Balance (FAB) test [44, 45].

### Data analysis

#### Creation of the lower extremity loss-of-function CIPN phenotypes

The following characteristics were used to create the lower extremity loss-of-function CIPN phenotypes: rating of worst pain in the past 24 h, loss of light touch sensation, loss of cold sensation, loss of pain sensation, vibration threshold, TUG score, and FAB score. For light touch (9 sites), cold (4 sites), and pain (9 sites), the number of sites with loss of each sensation, across all the sites that were tested, was summed. For vibration, the mean score across the three sites was calculated. For the TUG and FAB, mean scores were used in the analysis.

#### Latent profile analysis (LPA)

Unconditional LPA was used to identify subgroups of survivors with distinct lower extremity loss-of-function CIPN profiles (i.e., latent classes). Estimation was carried out with full information maximum likelihood with standard errors and a Chi-square test that are robust to nonnormality and non-independence of observations (“estimator = MLR”). Model fit was evaluated to identify the best solution that characterized the observed latent class structure with the Bayesian Information Criterion (BIC), the Vuong-Lo-Mendell-Rubin likelihood ratio test (VLMR) for the *K* vs. *K*−1 model, entropy, and latent class percentages that were large enough to be reliable (i.e., likely to replicate in new samples) [46, 47]. Missing data were accommodated with the use of the expectation–maximization (EM) algorithm [48]. Mixture models, like LPA, are known to produce solutions at local maxima. Therefore, our models were fit with 1000 to 2400 random starts. This approach ensured that the estimated model was replicated many times and was not due to a local maximum. Estimation was done with Mplus Version 7.2 [46].

#### Additional analyses

Descriptive statistics and frequency distributions were calculated for survivors’ demographic and clinical characteristics using IBM SPSS Statistics version 29 (IBM Corporation, Armonk, New York). Differences between the two loss of function profiles in demographic and clinical characteristics, pain characteristics, pain interference scores, and pain quality scores were evaluated using parametric and nonparametric tests. Using Stata 15.1 (StataCorp, College Station, TX), multivariable logistic regression analysis was done to assess the relationships between select demographic and clinical characteristics that differed in the bivariable analyses and CIPN profile membership. A *p*-value of < 0.05 was considered statistically significant.

## Results

### Results of the LPA

As shown in Table 1, the two-class solution was selected because the BIC for that solution was lower than the BIC for the 1-class solution. In addition, the VLMR was significant for the 2-class solution, indicating that two classes fit the data better than one class. Although the BIC was smaller for the 3-class than for the 2-class solution, the VLMR was not significant for the 3-class solution, indicating that too many classes were extracted. In addition, the 3-class solution included a very small predicted class (only 27 predicted cases; 6.7% of the sample), raising the concern that the solution may not generalize to other samples. As shown in Table 2, based on the scores for the subjective and objective sensory measures, the classes were named less severe loss of LE Function (76.5%, *n* = 310; less severe) and more severe loss of LE Function (23.5%, *n* = 95; more severe).

**Table 1:** Latent profile solutions and fit indices lower extremity loss of function classes

Lower extremity loss of function latent profile analysis					
Model	LL	AIC	BIC	Entropy	VLMR
1 Class	−5328.46	10,684.91	10,740.97	n/a	n/a
2 Class^a^	−5145.93	10,335.85	10,423.94	0.83	365.06^**^
3 Class	−5056.26	10,172.51	10,292.63	0.89	NS

Baseline entropy and VLMR are not applicable for the one-class solution

^*^p <.01; ^**^p <.001

^a^The 2-class solution was selected because the BIC for that solution was lower than the BIC for the 1-class solution. In addition, the VLMR was significant for the 2-class solution, indicating that two classes fit the data better than one class. Although the BIC was smaller for the 3-class than for the 2-class solution, the VLMR for 3 classes was not significant, indicating that too many classes had been extracted

Abbreviations: *AIC* Akaike’s Information Criterion, *BIC* Bayesian Information Criterion, *LL* log-likelihood, *n/a* not applicable, *NS* not significant, *VLMR* Vuong-Lo-Mendell-Rubin likelihood ratio test for the *K* vs. *K*−1 model

**Table 2:** Sensory profile measures for the lower extremity loss of function classes

Sensory profile measure*^+^	Less severe loss of LE function 76.5% (*n* = 310)	More severe loss of LE function 23.5% (*n* = 95)
	Mean (SE)	Mean (SE)
Worst pain (0 to 10)	5.8 (0.15)	6.9 (0.34)
Loss of light touch sensation (0 to 9)^a^	1.3 (0.15)	5.2 (0.52)
Loss of cold sensation (0 to 4)^b^	2.0 (0.08)	3.0 (0.12)
Loss of pain sensation (0 to 9)^c^	3.0 (0.13)	4.9 (0.28)
Vibration threshold (volts)^d^ (normal = < 15 V, mild = 20 to 25 V, moderate = 20 to 25 V, severe ≥ 25 V)	24.0 (0.09)	37.3 (0.14)
Timed get up and go test (> 13.5 s)	7.2 (0.14)	9.3 (0.52)
Fullerton Advanced Balance test (≤ 25 is associated with a higher risk of falls)	35.0 (0.04)	27.2 (0.15)

Abbreviation: *LE *lower extremity, *SE* standard error

^*^When available, the clinically meaningful cut-point score is provided in parentheses next to the characteristic

^+^Changes in sensation are reported for the dominant extremity

^a^Lower extremity sites for light touch were as follows: pad of great toe, pad of 3rd toe, pad of 5th toe, base of heel, metocarpophalangeal (MP) joint of great toe, MP joint of 3rd toe, MP joint of 5th toe, midway along the tibia, patella

^b^Lower extremity sites for cold were as follows: top of great toe at 1 st MP joint, pad of great toe, dorsum of foot midpoint, medial malleolus

^c^Lower extremity sites for pain were as follows: pad of great toe, pad of 3rd toe, pad of 5th toe, base of heel, MP joint of great toe, MP joint of 3rd toe, MP joint of 5th toe, midway along the tibia, patella

^d^Lower extremity sites for vibration were as follows: dorsal ipsilateral joint of great toe, medial malleolus, patella

### Differences in demographic and clinical characteristics

As shown in Table 3, survivors in the more severe class were older, more likely to be male, more likely to live alone, less likely to be employed, and more likely to have a lower annual household income. In terms of clinical characteristics (Table 4), survivors in the more severe class had a lower functional status score, a higher body mass index, and a higher number of comorbidities and a worse comorbidity profile, a lower AUDIT score, a longer time since their cancer diagnosis, and were receiving fewer current cancer treatments. In addition, they were more likely to report a smoking history, less likely to exercise on a regular basis, and more likely to self-report diagnoses of depression, high blood pressure, heart disease, diabetes, lung disease, and kidney disease.

**Table 3:** Differences in demographic characteristics between the lower extremity loss-of-function classes

Characteristic	Less severe loss of LE function 76.5% (n = 310)	More severe loss of LE function 23.5% (*n *= 95)	Test, *p*-value
	Mean (SD)	Mean (SD)	
Age (years)	59.4 (10.5)	66.1 (9.0)	*t* = −5.63, *p* < 0.001
Education (years)	16.5 (2.7)	15.9 (3.0)	*t* = 1.86, *p* = 0.064
	% (*n*)	% (*n*)	
Female	90.0 (278)	74.7 (71)	FE, *p* < 0.001
Married/partnered (% yes)	63.0 (191)	53.8 (49)	FE, *p* = 0.141
Lives alone (% yes)	26.6 (81)	39.4 (37)	FE, *p* = 0.020
Employed (% yes)	47.7 (148)	23.4 (22)	FE, *p* < 0.001
Race or ethnicity White Asian/Pacific Islander Black Hispanic/mixed/other	76.5 (237) 8.4 (26) 4.2 (13) 11.0 (34)	80.0 (76) 2.1 (2) 8.4 (8) 9.5 (9)	*Χ*^2^ = 6.93, *p* = 0.074
Annual household income < $30,000 $30,000–$69,999 $70,000–$99,999 > $100,000	19.5 (57) 18.8 (55) 18.4 (54) 43.3 (127)	37.3 (31) 28.9 (24) 9.6 (8) 24.1 (20)	U, *p* < 0.001
Child care responsibilities (% yes)	14.1 (43)	8.4 (8)	FE, *p* = 0.163
Adult care responsibilities (% yes)	3.2 (9)	3.3 (3)	FE,* p* = 1.000

Abbreviations: *FE* Fisher’s exact test, *LE* lower extremity, *SD* standard deviation, *U* Mann–Whitney *U* test

**Table 4:** Differences in clinical characteristics between the lower extremity loss-of-function classes

Characteristic	Less severe loss of LE function (1) 76.5% (*n* = 310)	More severe loss of LE function (2) 23.5% (*n* = 95)	Test, *p*-value
	Mean (SD)	Mean (SD)	
Karnofsky performance status score	84.3 (9.7)	78.9 (11.2)	*t* = 4.16, *p* < 0.001
Body mass index (kg/m^2^)	26.2 (5.2)	27.8 (6.5)	*t* = −2.10, *p* = 0.038
Number of comorbidities	1.8 (1.4)	2.6 (1.7)	*t = −*4.24, *p* < 0.001
SCQ score	3.8 (3.2)	5.5 (3.7)	*t* = −3.99, *p *< 0.001
AUDIT score	2.4 (2.3)	1.9 (2.0)	*t* = 2.00, *p* = 0.046
Years since cancer diagnosis	4.5 (4.6)	5.8 (5.2)	*t* = −2.18, *p* = 0.031
Number of prior cancer treatments	3.2 (1.0)	3.0 (1.0)	*t* = 1.65, *p* = 0.099
Number of current cancer treatments	0.5 (0.6)	0.3 (0.4)	*t* = 3.12, *p* = 0.002
Number of metastatic sites (out of 7)	0.7 (0.8)	0.8 (0.8)	*t* = −0.40, *p* = 0.687
Number of metastatic sites without lymph node involvement	0.2 (0.6)	0.2 (0.5)	*t* = 0.07, *p* = 0.944
Only platinum			
Total dose of cisplatin (mg/m^2^) Total dose of carboplatin (mg/m^2^) Total dose of oxaliplatin (mg/m^2^)	235.6 (109.0) 1615.0 (1037.6) 824.6 (248.8)	297.7 (155.0) 1252.3 (598.5) 779.6 (237.4)	*t* = −1.31, *p* = 0.200 *t* = 0.54, *p* = 0.607 *t *= 0.62, *p* = 0.538
Only taxane			
Total dose of taxol (mg/m^2^) Total dose of docetaxel (mg/m^2^) Total dose of abraxane (mg/m^2^)	802.7 (255.1) 320.2 (135.0) 659.9 (388.9)	1060.9 (1580.0) 296.6 (103.5) ––	*t* = −0.83, *p* = 0.414 *t *= 0.44, *p* = 0.665 –
Both platinum and taxane			
Total dose of cisplatin (mg/m^2^) Total dose of carboplatin (mg/m^2^) Total dose of oxaliplatin (mg/m^2^) Total dose of taxol (mg/m^2^) Total dose of docetaxel (mg/m^2^) Total dose of abraxane (mg/m^2^)	395.4 (250.5) 1808.9 (687.2) 74.6 (51.7) 987.2 (378.0) 417.3 (222.6) 1034.0 (594.7)	221.3 (300.2) 2052.6 (865.9) 86.1 –– 1117.8 (426.5) 382.7 (73.5) 703.5 ––	*t* = 1.23 *p* = 0.231 *t *= −1.41, *p* = 0.160 – *t* = −1.26, *p* = 0.212 *t* = 0.37, *p* = 0.712 ––
	% (n)	% (n)	
Previous or current history of smoking	35.0 (108)	48.9 (46)	FE, *p* = 0.016
Exercise on a regular basis (% yes)	88.4 (274)	76.6 (72)	FE, *p* = 0.007
Born prematurely (% yes)	6.7 (19)	5.7 (5)	FE, *p* = 1.000
Surgery on legs (% yes)	24.4 (74)	26.6 (25)	FE, *p* = 0.683
Surgery on feet (% yes)	15.7 (48)	20.4 (19)	FE, *p* = 0.342
Injury to legs (% yes)	22.1 (67)	26.9 (25)	FE, *p *= 0.400
Injury to feet (% yes)	26.3 (79)	32.6 (30)	FE, *p* = 0.287
Comorbid conditions (% yes)			
Osteoarthritis Back pain Depression High blood pressure Heart disease Diabetes Lung disease Anemia or blood disease Ulcer or stomach disease Kidney disease Liver disease Rheumatoid arthritis	28.7 (89) 34.5 (107) 20.3 (63) 22.9 (71) 6.1 (19) 4.5 (14) 3.5 (11) 4.8 (15) 2.3 (7) 1.3 (4) 2.3 (7) 2.6 (8)	36.8 (35) 33.7 (32) 33.7 (32) 37.9 (36) 12.6 (12) 10.5 (10) 9.5 (9) 8.4 (8) 6.3 (6) 6.3 (6) 6.3 (6) 4.2 (4)	FE, *p* = 0.161 FE, *p* = 0.902 FE, *p* = 0.009 FE, *p* = 0.005 FE, *p* = 0.047 FE, *p* = 0.044 FE, *p* = 0.029 FE, *p* = 0.206 FE, *p* = 0.087 FE, *p* = 0.013 FE, *p* = 0.087 FE, *p* = 0.487
Type of cancer Breast Colon Lung Ovarian Other	57.1 (177) 9.4 (29) 1.6 (5) 10.6 (33) 21.3 (66)	45.3 (43) 11.6 (11) 2.1 (2) 10.5 (10) 30.5 (29)	*Χ*^2^ = 4.99, *p* = 0.289
Any metastatic disease (% yes)	59.3 (182)	65.2 (60)	FE, *p* = 0.332
Type of chemotherapy regimen			
Only platinum Only taxane Both platinum and taxane	19.7 (61) 49.0 (152) 31.3 (97)	33.7 (32) 37.9 (36) 28.4 (27)	*Χ*^2^ = 8.35, *p* = 0.015 1 < 2 NS NS
Patients who had a dose reduction or delay due to neuropathy	13.4 (40)	16.9 (15)	FE, *p* = 0.489

Abbreviations:* AUDIT* Alcohol Use Disorders Identification Test, *FE* Fisher’s exact test, *kg* kilograms, *LE* lower extremity, *m*^2^ meters squared, *mg* milligrams, *NS* not significant, *SCQ* Self-Administered Comorbidity Questionnaire, *SD* standard deviation

Of note, no differences were found between the two groups in types of cancer, number of prior cancer treatments, number of metastatic sites or presence of metastatic disease, doses of chemotherapy drugs received, and number of dose reductions or delays due to CIPN. In terms of the chemotherapy regimen, of the three possible pairwise contrasts, the only one that was significant was the comparison for the only platinum regimen. Survivors in the more severe class were more likely to receive an only platinum-containing regimen.

### Association between demographic and clinical characteristics and membership in the more severe loss of lower extremity function class

As shown in Table 5, in the multivariable logistic regression analysis that included select demographic and clinical characteristics that differed between the two profiles (N.B., SCQ score was used instead of the number of comorbidities to account for comorbidity burden; annual income was not included in the analysis because of a large amount of missing data), age, gender, and KPS score were the only characteristics that remained significant. Patients who were older, male, and had a lower functional status score were more likely to be in the more severe class.

**Table 5:** Multivariable logistic regression analysis of associations among demographic and clinical characteristics and membership in the more severe loss of lower extremity function class (*n* = 370)

Characteristics	Adjusted odds ratio	Standard error	95% CI	*Z*	*p*-value
Age	1.11	0.02	1.06, 1.15	5.20	< 0.001
Male gender	3.76	2.04	1.30, 10.92	2.44	0.015
Living alone	1.35	0.42	0.73, 2.49	0.96	0.337
Being employed	0.61	0.21	0.31, 1.22	−1.40	0.162
KPS score	0.94	0.02	0.91, 0.97	−3.89	< 0.001
Body mass index	1.03	0.03	0.97, 1.08	0.94	0.349
SCQ score	1.07	0.05	0.98, 1.16	1.44	0.151
AUDIT score	0.87	0.06	0.76, 1.01	−1.83	0.067
Years since cancer diagnosis	1.03	0.03	0.97, 1.09	0.88	0.378
Number of current cancer treatments	0.77	0.24	0.42, 1.43	−0.83	0.405
Previous or current history of smoking	1.20	0.36	0.67, 2.15	0.61	0.539
Exercise on a regular basis	0.70	0.26	0.33, 1.46	−0.95	0.340
Chemotherapy regimen					
Only platinum (Ref) Only taxane Both platinum and taxane	1.00 0.80 0.68	0.40 0.34	0.30, 2.12 0.26, 1.80	−0.45 −0.77	0.650﻿ 0.442
Overall model fit: *X*^2^ = 104.23; *p* < 0.001; pseudo *R*^2^ = 0.2568					

Abbreviations: *AUDIT* Alcohol Use Disorders Identification Test, *CI* confidence interval, *KPS* Karnofsky Performance Status, *SCQ* Self-Administered Comorbidity Questionnaire

### Differences in pain characteristics

As shown in Table 6, except for hours per day in pain, survivors in the more severe class reported higher scores for all of the pain characteristics and pain interference scores. In addition, except for aching, survivors in the more severe class reported higher scores for all of the pain qualities, as well as for all of the PQAS subscale scores.

**Table 6:** Differences in pain characteristics between the lower extremity loss-of-function classes

Characteristic	Less severe loss of LE function 76.5% (n = 310)	More severe loss of LE function 23.5% (n = 95)	Statistic, *p-*value
	Mean (SD)	Mean (SD)	
Pain characteristics			
Duration of CIPN (years)	3.6 (4.0)	5.1 (4.9)	*t* = −2.81, *p* = 0.006
Pain now	3.4 (2.2)	4.3 (2.3)	*t* = −3.37, p = 0.001
Average pain	3.8 (2.1)	4.7 (2.1)	*t* = −3.84, *p* < 0.001
Days per week in pain	3.3 (3.0)	4.8 (2.8)	*t* = −4.74, *p* < 0.001
Hours per day in pain	14.6 (9.6)	16.2 (8.9)	*t* = −1.37, *p* = 0.171
Pain Interference Scale			
Balance	3.2 (2.9)	4.9 (3.2)	*t* = −4.71, p < 0.001
Walking ability	3.0 (2.9)	4.4 (3.2)	*t* = −3.76, *p* < 0.001
Enjoyment of life	2.6 (2.6)	3.8 (3.2)	*t *= −3.41, *p* < 0.001
Normal work	2.3 (2.6)	3.9 (3.2)	*t* = −4.18, *p* < 0.001
Sleep	2.4 (2.7)	3.7 (3.2)	*t* = −3.55, *p* < 0.001
General activity	2.2 (2.4)	4.0 (3.0)	*t* = −5.10, *p* < 0.001
Mood	2.1 (2.3)	3.3 (2.8)	*t *= −3.70, *p* < 0.001
Relations with other people	1.2 (2.0)	2.5 (2.8)	*t* = −4.06, *p* < 0.001
Sexual activity	0.7 (1.8)	1.7 (3.0)	*t* = −2.81, *p* = 0.006
Mean interference score	2.2 (2.0)	3.6 (2.5)	*t* = −4.73, *p* < 0.001
Pain Qualities Assessment Scale (PQAS) Scores			
Numb	5.1 (3.0)	6.4 (2.9)	*t* = −3.80, *p* < 0.001
Unpleasant	4.2 (2.4)	5.5 (2.4)	*t* = −4.82, *p* < 0.001
Tingling	4.0 (3.0)	5.4 (2.7)	*t* = −4.09, *p* < 0.001
Intense	2.9 (2.4)	4.4 (2.6)	*t* = −4.99, *p* < 0.001
Dull	3.0 (2.7)	3.6 (2.7)	*t* = −2.02, *p* = 0.044
Cramping	2.5 (3.0)	4.0 (3.5)	*t* = −3.60, *p* < 0.001
Electrical	2.3 (2.9)	3.3 (3.5)	*t* = −2.44, *p* = 0.016
Shooting	2.3 (2.8)	3.1 (3.3)	*t* = −2.30, *p* = 0.023
Sharp	2.1 (2.7)	3.2 (3.3)	*t *= −3.06, *p* = 0.003
Aching	2.1 (2.6)	2.7 (3.1)	*t* = −1.82, *p* = 0.071
Heavy	1.8 (2.5)	3.0 (3.2)	*t* = −3.10, *p* = 0.002
Cold	1.9 (2.8)	2.8 (2.9)	*t* = −2.76, *p* = 0.006
Radiating	1.8 (2.6)	2.9 (3.0)	*t* = −3.27, *p* = 0.001
Hot	1.8 (2.5)	2.7 (3.1)	*t* = −2.58, *p* = 0.011
Tender	1.8 (2.4)	2.4 (2.8)	*t* = −2.02, *p* = 0.046
Sensitive skin	1.6 (2.1)	2.4 (2.8)	*t* = −2.43, *p* = 0.016
Throbbing	1.6 (2.4)	2.3 (3.0)	*t* = −2.25, *p* = 0.026
Itchy	0.9 (1.8)	1.6 (2.6)	*t* = −2.59, *p* = 0.011
Intense—surface pain	3.0 (2.6)	4.2 (2.8)	*t* = −3.63, *p* < 0.001
Intense—deep pain	3.1 (2.7)	4.1 (3.1)	*t* = −2.90, *p* = 0.004
PQAS subscale—paroxysmal	2.0 (2.1)	3.1 (2.6)	*t* = −3.52, *p* = 0.001
PQAS subscale—surface	2.7 (1.6)	3.7 (1.8)	*t* = −5.24, *p* < 0.001
PQAS subscale—deep	2.2 (1.9)	3.1 (2.3)	*t* = −3.53, *p* = 0.001

Abbreviations: *CIPN* chemotherapy-induced peripheral neuropathy, *LE* lower extremity, *SD* standard deviation

## Discussion

This study is the first to use subjective and objective measures to identify two groups of survivors with distinct CIPN profiles. These survivors differed in their ratings of pain severity, as well as in their loss of primary sensations and balance problems. Of note, the more severe class, that included approximately 25% of the sample, had pain intensity scores in the severe range and clinically meaningful decrements in several measures of lower extremity function. In addition, their FAB test scores approached the cutpoint associated with an increased risk of falls. Because previous studies that used cluster analysis to identify subgroups of patients with distinct “pain profiles” used different measures and did not include patients or survivors with CIPN [11, 12], direct comparisons cannot be performed. However, the current findings suggest that different CIPN phenotypes exist and that mechanistic studies are warranted.

### Demographic and clinical characteristics

While in the bivariable analyses, numerous demographic (Table 3) and clinical (Table 4) characteristics differed between the two classes, in the multivariable analysis (Table 5), being older, being male, and having a lower functional status were the only characteristics associated with a significant increase in the odds of being in the more severe class. The finding of older age being associated with membership in the more severe class is consistent with previous reports [49–51]. As noted in several reviews [52–54], aging is associated with changes in organ function, body composition, significant multimorbidity, and increased use of multiple medications that can impact drug metabolism and increase the risk for adverse effects associated with the administration of chemotherapy. While controversy exists on the magnitude of these age-related physiologic changes in pharmacokinetics and pharmacodynamics and whether dosage modifications are warranted in older adults [54], in the current study, no between-group differences were found in the doses of platinum and/or taxane compounds that the survivors received. Given the increased interest in accelerated biological aging in oncology patients [55, 56], future studies should include measures of epigenetic aging and its association with the development of CIPN.

In a recent study of risk factors for CIPN in patients with lung cancer [57], the authors noted that findings regarding sex differences in CIPN are inconclusive because most of the studies were done in patients with breast cancer. In the current study, being male was associated with a 3.76-fold increase in the odds of being in the more severe class. While additional research is warranted on sex differences in the occurrence and severity of CIPN in oncology patients and survivors, evidence from preclinical models demonstrates sexual dimorphism in the mechanisms of and treatments for CIPN [58–60]. As noted in a review of sex differences in drug effects and/or toxicity in oncology [61], these sexually dimorphic effects may be related to differences in immune function; hormonal mediators like estradiol and androgens; and social sex behaviors (e.g., smoking and alcohol use). Equally important, additional research is warranted on potential interactions between sex differences in adverse effects and perceptions and responses to pain [62].

Similar to an evaluation of sex differences, findings regarding an association with functional status and the occurrence of CIPN are limited and inconclusive [50, 51, 63]. In the current study, the differences in KPS scores between the less severe (84.3) and more severe (78.9) classes represent not only statistically significant but also clinically meaningful decrements in functional status (Cohen’s *d* = 0.52 [64]) [65, 66]. While this association may be linked to older age and a higher level of multimorbidity in the more severe class, it warrants evaluation by clinicians because an increased risk of falls is associated with poorer functional status in patients with CIPN. This finding is corroborated by the lower FAB scores in this class [67].

While in the bivariable analysis, a higher percentage of patients who had received only a platinum-containing regimen were in the more severe class, type of chemotherapy regimen was not significant in the multivariable analysis. In addition, no between-group differences were found in cancer diagnoses, total doses of chemotherapy received, and dose reduction or delay due to chemotherapy. Given the inconsistent findings across studies, additional longitudinal studies are needed to assess these characteristics in patients during active treatment and into survivorship.

### Loss of large fiber function and CIPN

In terms of the loss of large fiber function measures (i.e., light touch and vibration), compared to the less severe class, survivors in the more severe class had four times the number of sites that lost light touch sensation. In addition, their vibration thresholds were 1.5 times higher and were well above the severe cutoff score of > 25 V [40]. Of note, the between-class ratings of numbness (a subjective measure of loss of large fiber function) demonstrated not only a statistically significant difference but a clinically meaningful difference in its intensity (i.e., 5.1 versus 6.4, Cohen’s *d* = −0.42 [64]) [65, 66]. While our previous study evaluated only simple correlations between average pain intensity scores and loss of light touch sensations, decrements in vibration thresholds, and increased severity of numbness to demonstrate the involvement of large fiber loss in patients with CIPN [20], the current study confirms this association using a more robust analytic method (i.e., latent variable modeling) that integrates the three measures of large fiber function. In addition, the FAB scale, that measures both static and dynamic balance under different sensory conditions, provides indirect information on multiple sensory and motor systems (i.e., visual, vestibular, and proprioceptive systems) [44, 45]. Given that large-diameter myelinated fibers (i.e., Aα and Aβ fibers) are involved in proprioception [68] and that the FAB test results are significantly worse in the more severe class, this provides additional support for the hypothesis that large fiber loss is involved in the CIPN phenotype.

### Potential involvement of C-tactile fibers in CIPN

As noted in the Introduction, stimulation of a subgroup of C fibers known as C-tactile fibers is thought to contribute to the perception of pleasantness during gentle stroking of hairy skin (i.e., “affective touch”) [22–24]. Stimulation of these fibers in humans involves the use of a soft watercolor brush made of fine smooth goat hair that is stroked at very low and uniform velocities. Germaine to this discussion, evidence from a preclinical study suggests that these fibers play a role in the pathophysiology of tactile allodynia [25]. While an objective measure of C-tactile fibers was not done in the current study, it is known that some patients with CIPN report tactile allodynia (e.g., burning, tender sensations from non-noxious stimuli) and describe the sensation as extremely unpleasant [26]. In the current study, significant between-group differences in survivors’ ratings of the severity of sensitive skin (i.e., “Tell us how sensitive the skin in your feet has been to light touch or clothing rubbing against it over the past week. Words used to describe sensitive skin include ‘like sunburned skin’ and ‘raw skin’.”) and unpleasant (i.e., “Now that you have told us the different types of pain sensations you have felt, we want you to tell us overall how unpleasant your pain in your feet has been to you in the past week, Words used to describe unpleasant pain include ‘annoying,’ ‘bothersome,’ ‘miserable,’ and ‘intolerable’.”) support the hypothesis that these fibers may be damaged by neurotoxic chemotherapy (see PQAS ratings on Table 6). To evaluate the role of C-tactile fiber activity in CIPN, future studies should incorporate an objective assessment of these fibers, as well as tactile allodynia.

### Limitations

While this study describes a novel phenotypic characterization of CIPN and provides evidence to support the role of large-diameter and C-tactile fibers in CIPN, several limitations warrant consideration. While the sample size was sufficient for LPA, larger samples may reveal additional profiles and/or additional risk factors. The sample was primarily female, White, and well educated. Given that various social determinants of health may influence the stage of the disease at the time of diagnosis and associated treatments, as well as an increased likelihood of multimorbidity [69], future studies need to include survivors with more diverse backgrounds. In addition, because survivors need to attend an in-person testing session, selection bias may be present. Given that the objective measures used in this study were designed for clinical use, more detailed characterization of neural activity may provide additional insights into the role of large-diameter and C-tactile fibers in CIPN.

## Conclusions and recommendations for future research

This study’s findings suggest that distinct “CIPN phenotypes” can be identified in cancer survivors. The survivors in the Morse Severe class experienced CIPN for 1.5 years longer than the Less Severe class (i.e., 5.1 versus 3.6 years, respectively). While both classes had persistent pain, longitudinal studies are warranted to determine if the CIPN phenotype worsens over time. In addition, studies are warranted to determine the underlying mechanisms for a worsening phenotype (e.g., epigenetic changes). Detailed phenotyping and molecular characterization of various CIPN phenotypes will lead to the development and testing of targeted and personalized interventions.

## Data Availability

Data is available from the corresponding author following the completion of a data sharing agreement with the University of California, San Francisco.

## References

[1] Kandula T, Farrar MA, Kiernan MC, Krishnan AV, Goldstein D, Horvath L et al (2017) Neurophysiological and clinical outcomes in chemotherapy-induced neuropathy in cancer. Clin Neurophysiol 128(7):1166–75. 10.1016/j.clinph.2017.04.009 28511129 10.1016/j.clinph.2017.04.009

[2] Brewer JR, Morrison G, Dolan ME, Fleming GF (2016) Chemotherapy-induced peripheral neuropathy: current status and progress. Gynecol Oncol 140(1):176–183. 10.1016/j.ygyno.2015.11.011 26556766 10.1016/j.ygyno.2015.11.011 PMC4698212

[3] Postma TJ, Heimans JJ (2000) Grading of chemotherapy-induced peripheral neuropathy. Ann Oncol 11(5):509–13. 10.1023/a:1008345613594 10907941 10.1023/a:1008345613594

[4] Visovsky C, Collins M, Abbott L, Aschenbrenner J, Hart C (2007) Putting evidence into practice: evidence-based interventions for chemotherapy-induced peripheral neuropathy. Clin J Oncol Nurs 11(6):901–913. 10.1188/07.CJON.901-913 18063548 10.1188/07.CJON.901-913

[5] Windebank AJ, Grisold W (2008) Chemotherapy-induced neuropathy. J Peripher Nerv Syst 13(1):27–46. 10.1111/j.1529-8027.2008.00156.x 18346229 10.1111/j.1529-8027.2008.00156.x

[6] Wolf S, Barton D, Kottschade L, Grothey A, Loprinzi C (2008) Chemotherapy-induced peripheral neuropathy: prevention and treatment strategies. Eur J Cancer 44(11):1507–15. 10.1016/j.ejca.2008.04.018 18571399 10.1016/j.ejca.2008.04.018

[7] Vanotti A, Osio M, Mailland E, Nascimbene C, Capiluppi E, Mariani C (2007) Overview on pathophysiology and newer approaches to treatment of peripheral neuropathies. CNS Drugs 21(Suppl 1):3–12. 10.2165/00023210-200721001-00002 17696588 10.2165/00023210-200721001-00002

[8] Attal N, Bouhassira D, Baron R, Dostrovsky J, Dworkin RH, Finnerup N et al (2011) Assessing symptom profiles in neuropathic pain clinical trials: can it improve outcome? Eur J Pain 15(5):441–3. 10.1016/j.ejpain.2011.03.005 21458336 10.1016/j.ejpain.2011.03.005

[9] Baron R, Dickenson AH (2014) Neuropathic pain: precise sensory profiling improves treatment and calls for back-translation. Pain 155(11):2215–2217. 10.1016/j.pain.2014.08.021 25168667 10.1016/j.pain.2014.08.021

[10] Baron R, Forster M, Binder A (2012) Subgrouping of patients with neuropathic pain according to pain-related sensory abnormalities: a first step to a stratified treatment approach. Lancet Neurol 11(11):999–1005. 10.1016/S1474-4422(12)70189-8 23079556 10.1016/S1474-4422(12)70189-8

[11] Baron R, Maier C, Attal N, Binder A, Bouhassira D, Cruccu G et al (2017) Peripheral neuropathic pain: a mechanism-related organizing principle based on sensory profiles. Pain 158(2):261–72. 10.1097/j.pain.0000000000000753 27893485 10.1097/j.pain.0000000000000753 PMC5266425

[12] Freeman R, Baron R, Bouhassira D, Cabrera J, Emir B (2014) Sensory profiles of patients with neuropathic pain based on the neuropathic pain symptoms and signs. Pain 155(2):367–76. 10.1016/j.pain.2013.10.023 24472518 10.1016/j.pain.2013.10.023

[13] Attal N, Fermanian C, Fermanian J, Lanteri-Minet M, Alchaar H, Bouhassira D (2008) Neuropathic pain: are there distinct subtypes depending on the aetiology or anatomical lesion? Pain 138(2):343–53. 10.1016/j.pain.2008.01.006 18289791 10.1016/j.pain.2008.01.006

[14] Tavee J (2019) Nerve conduction studies: basic concepts. Handb Clin Neurol 160:217–224. 10.1016/b978-0-444-64032-1.00014-x 31277849 10.1016/B978-0-444-64032-1.00014-X

[15] Woolf CJ, Ma Q (2007) Nociceptors--noxious stimulus detectors. Neuron 55(3):353–364. 10.1016/j.neuron.2007.07.016 17678850 10.1016/j.neuron.2007.07.016

[16] Boehmerle W, Huehnchen P, Peruzzaro S, Balkaya M, Endres M (2014) Electrophysiological, behavioral and histological characterization of paclitaxel, cisplatin, vincristine and bortezomib-induced neuropathy in C57Bl/6 mice. Sci Rep 4:6370. 10.1038/srep06370 25231679 10.1038/srep06370 PMC5377307

[17] Krarup-Hansen A, Helweg-Larsen S, Schmalbruch H, Rorth M, Krarup C (2007) Neuronal involvement in cisplatin neuropathy: prospective clinical and neurophysiological studies. Brain 130(Pt 4):1076–88. 10.1093/brain/awl356 17301082 10.1093/brain/awl356

[18] Dougherty PM, Cata JP, Cordella JV, Burton A, Weng HR (2004) Taxol-induced sensory disturbance is characterized by preferential impairment of myelinated fiber function in cancer patients. Pain 109(1–2):132–142 15082135 10.1016/j.pain.2004.01.021

[19] Saad M, Psimaras D, Tafani C, Sallansonnet-Froment M, Calvet JH, Vilier A et al (2016) Quick, non-invasive and quantitative assessment of small fiber neuropathy in patients receiving chemotherapy. J Neurooncol 127(2):373–80. 10.1007/s11060-015-2049-x 26749101 10.1007/s11060-015-2049-x

[20] Miaskowski C, Paul SM, Mastick J, Abrams G, Topp K, Smoot B et al (2019) Contribution of loss of large fiber function to pain in 2 samples of oncology patients. Clin J Pain 35(1):37–42. 10.1097/ajp.0000000000000649 30247200 10.1097/AJP.0000000000000649 PMC6309865

[21] Mansooralavi N, Khomula EV, Levine JD (2023) Duloxetine prevents bortezomib and paclitaxel large-fiber chemotherapy-induced peripheral neuropathy (LF-CIPN) in Sprague Dawley rats. Mol Pain 19:17448069231185694. 10.1177/17448069231185694 37338165 10.1177/17448069231185694 PMC10288414

[22] Vallbo AB, Olausson H, Wessberg J (1999) Unmyelinated afferents constitute a second system coding tactile stimuli of the human hairy skin. J Neurophysiol 81(6):2753–63. 10.1152/jn.1999.81.6.2753 10368395 10.1152/jn.1999.81.6.2753

[23] Löken LS, Wessberg J, Morrison I, McGlone F, Olausson H (2009) Coding of pleasant touch by unmyelinated afferents in humans. Nat Neurosci 12(5):547–548. 10.1038/nn.2312 19363489 10.1038/nn.2312

[24] Morrison I, Löken LS, Olausson H (2010) The skin as a social organ. Exp Brain Res 204(3):305–314. 10.1007/s00221-009-2007-y 19771420 10.1007/s00221-009-2007-y

[25] Seal RP, Wang X, Guan Y, Raja SN, Woodbury CJ, Basbaum AI et al (2009) Injury-induced mechanical hypersensitivity requires C-low threshold mechanoreceptors. Nature 462(7273):651–5. 10.1038/nature08505 19915548 10.1038/nature08505 PMC2810205

[26] Meregalli C, Monza L, Jongen JLM (2022) A mechanistic understanding of the relationship between skin innervation and chemotherapy-induced neuropathic pain. Front Pain Res 3:1066069. 10.3389/fpain.2022.1066069 10.3389/fpain.2022.1066069 PMC9792502 36582196

[27] Miaskowski C, Mastick J, Paul SM, Topp K, Smoot B, Abrams G et al (2017) Chemotherapy-induced neuropathy in cancer survivors. J Pain Symptom Manage 54(2):204–18 e2. 10.1016/j.jpainsymman.2016.12.342 28063866 10.1016/j.jpainsymman.2016.12.342 PMC5496793

[28] Babor TF, Higgins-Biddle JC, Saunders JB, Monteiro MG (2001) AUDIT: The Alcohol Use Disorders Identification Test: Guidelines for Use in Primary Care. World Health Organization, Geneva, Switzerland

[29] Karnofsky D (1977) Performance scale. Factors that influence the therapeutic response in cancer: a comprehensive treatise. Plenum Press, New York

[30] Sangha O, Stucki G, Liang MH, Fossel AH, Katz JN (2003) The self-administered comorbidity questionnaire: a new method to assess comorbidity for clinical and health services research. Arthritis Rheum 49(2):156–163. 10.1002/art.10993 12687505 10.1002/art.10993

[31] Posternak V, Dunn LB, Dhruva A, Paul SM, Luce J, Mastick J et al (2016) Differences in demographic, clinical, and symptom characteristics and quality of life outcomes among oncology patients with different types of pain. Pain 157(4):892–900. 10.1097/j.pain.0000000000000456 26683234 10.1097/j.pain.0000000000000456 PMC4939771

[32] Langford DJ, Schmidt B, Levine JD, Abrams G, Elboim C, Esserman L et al (2015) Preoperative breast pain predicts persistent breast pain and disability after breast cancer surgery. J Pain Symptom Manage 49(6):981–94. 10.1016/j.jpainsymman.2014.11.292 25527442 10.1016/j.jpainsymman.2014.11.292 PMC4470873

[33] Downie WW, Leatham PA, Rhind VM, Wright V, Branco JA, Anderson JA (1978) Studies with pain rating scales. Ann Rheum Dis 37(4):378–381 686873 10.1136/ard.37.4.378 PMC1000250

[34] Jensen MP, Dworkin RH, Gammaitoni AR, Olaleye DO, Oleka N, Galer BS (2005) Assessment of pain quality in chronic neuropathic and nociceptive pain clinical trials with the Neuropathic Pain Scale. J Pain 6(2):98–106 15694876 10.1016/j.jpain.2004.11.002

[35] Jensen MP, Gammaitoni AR, Olaleye DO, Oleka N, Nalamachu SR, Galer BS (2006) The pain quality assessment scale: assessment of pain quality in carpal tunnel syndrome. J Pain 7(11):823–832. 10.1016/j.jpain.2006.04.003 17074624 10.1016/j.jpain.2006.04.003

[36] Bell-Krotoski JA. Sensibility testing with the Semmes-Weinstein monofilaments. 5th ed. Rehabilitation of the hand and upper extremity. St Louis: Mosby, Inc.; 2002.

[37] Birke JA, Sims DS (1986) Plantar sensory threshold in the ulcerative foot. Lepr Rev 57(3):261–267. 10.5935/0305-7518.19860028 3784758 10.5935/0305-7518.19860028

[38] Birke JA, Brandsma JW, Schreuders TA, Piefer A (2000) Sensory testing with monofilaments in Hansen’s disease and normal control subjects. Int J Lepr Other Mycobact Dis 68(3):291–298 11221092

[39] Massy-Westropp N (2002) The effects of normal human variability and hand activity on sensory testing with the full Semmes-Weinstein monofilaments kit. J Hand Ther 15(1):48–52 11866352 10.1053/hanthe.2002.v15.01548

[40] Medakkel AA, Sheela P (2018) Vibration perception threshold values and clinical symptoms of diabetic peripheral neuropathy. J Clin Diagn Res 12(5):LC20–LC23

[41] Papanas N, Ziegler D (2011) New diagnostic tests for diabetic distal symmetric polyneuropathy. J Diabetes Complicat 25(1):44–51. 10.1016/j.jdiacomp.2009.09.006 10.1016/j.jdiacomp.2009.09.006 19896871

[42] Viswanathan V, Snehalatha C, Seena R, Ramachandran A (2002) Early recognition of diabetic neuropathy: evaluation of a simple outpatient procedure using thermal perception. Postgrad Med J 78(923):541–542. 10.1136/pmj.78.923.541 12357015 10.1136/pmj.78.923.541 PMC1742498

[43] Mathias S, Nayak US, Isaacs B (1986) Balance in elderly patients: the “get-up and go” test. Arch Phys Med Rehabil 67(6):387–389 3487300

[44] Hernandez D, Rose DJ (2008) Predicting which older adults will or will not fall using the Fullerton Advanced Balance scale. Arch Phys Med Rehabil 89(12):2309–2315. 10.1016/j.apmr.2008.05.020 18976981 10.1016/j.apmr.2008.05.020

[45] Rose DJ, Lucchese N, Wiersma LD (2006) Development of a multidimensional balance scale for use with functionally independent older adults. Arch Phys Med Rehabil 87(11):1478–1485. 10.1016/j.apmr.2006.07.263 17084123 10.1016/j.apmr.2006.07.263

[46] Muthen LK, Muthen BO (1998–2020) Mplus User’s Guide (8th ed.), 8th ed. Muthen & Muthen, Los Angeles, CA

[47] Nylund KL, Asparoutiov T, Muthén BO (2007) Deciding on the number of classes in latent class analysis and growth mixture modeling:: A Monte Carlo simulation study. Struct Equ Modeling 14(4):535–69. 10.1080/10705510701575396

[48] Muthen B, Shedden K (1999) Finite mixture modeling with mixture outcomes using the EM algorithm. Biometrics 55(2):463–469. 10.1111/j.0006-341x.1999.00463.x 11318201 10.1111/j.0006-341x.1999.00463.x

[49] Molassiotis A, Cheng HL, Leung KT, Li YC, Wong KH, Au JSK et al (2019) Risk factors for chemotherapy-induced peripheral neuropathy in patients receiving taxane- and platinum-based chemotherapy. Brain Behav 9(6):e01312. 10.1002/brb3.1312 31063261 10.1002/brb3.1312 PMC6576180

[50] Kleckner IR, Jusko TA, Culakova E, Chung K, Kleckner AS, Asare M et al (2021) Longitudinal study of inflammatory, behavioral, clinical, and psychosocial risk factors for chemotherapy-induced peripheral neuropathy. Breast Cancer Res Treat 189(2):521–32. 10.1007/s10549-021-06304-6 34191201 10.1007/s10549-021-06304-6 PMC8668235

[51] Xu H, Li H, Fan Y, Wang Y, Li Z, Zhou L et al (2025) Analysis of factors influencing chemotherapy-induced peripheral neuropathy in breast cancer patients using a random forest model. Breast 81:104457. 10.1016/j.breast.2025.104457 40245641 10.1016/j.breast.2025.104457 PMC12144932

[52] He X, Clarke SJ, McLachlan AJ (2011) Clinical pharmacology of chemotherapy agents in older people with cancer. Curr Gerontol Geriatr Res 2011:628670. 10.1155/2011/628670 21845189 10.1155/2011/628670 PMC3154497

[53] Sekine I, Fukuda H, Kunitoh H, Saijo N (1998) Cancer chemotherapy in the elderly. Jpn J Clin Oncol 28(8):463–473. 10.1093/jjco/28.8.463 9769779 10.1093/jjco/28.8.463

[54] Hurria A, Lichtman SM (2007) Pharmacokinetics of chemotherapy in the older patient. Cancer Control 14(1):32–43 17242669 10.1177/107327480701400105

[55] Abraham S, Parekh J, Lee S, Afrin H, Rozenblit M, Blenman KRM et al (2025) Accelerated aging in cancer and cancer treatment: current status of biomarkers. Cancer Med 14(9):e70929. 10.1002/cam4.70929 40322791 10.1002/cam4.70929 PMC12051034

[56] Wang S, El Jurdi N, Thyagarajan B, Prizment A, Blaes AH (2024) Accelerated aging in cancer survivors: cellular senescence, frailty, and possible opportunities for interventions. Int J Mol Sci. 10.3390/ijms25063319 38542292 10.3390/ijms25063319 PMC10970400

[57] von Itzstein MS, Rashdan S, Dahlberg SE, Gerber DE, Sandler AB, Schiller JH et al (2025) Incidence and correlates of high-grade chemotherapy-induced peripheral neuropathy in patients with lung cancer. Oncologist. 10.1093/oncolo/oyaf036 10.1093/oncolo/oyaf036 PMC11950918 40152312

[58] Cao Y, Jiang W, Yan F, Pan Y, Gei L, Lu S et al (2024) Sex differences in PD-L1-induced analgesia in paclitaxel-induced peripheral neuropathy mice depend on TRPV1-based inhibition of CGRP. CNS Neurosci Ther 30(7):e14829. 10.1111/cns.14829 38961264 10.1111/cns.14829 PMC11222069

[59] Staurengo-Ferrari L, Bonet IJM, Araldi D, Green PG, Levine JD (2022) Neuroendocrine stress axis-dependence of duloxetine analgesia (anti-hyperalgesia) in chemotherapy-induced peripheral neuropathy. J Neurosci 42(3):405–415. 10.1523/jneurosci.1691-21.2021 34880120 10.1523/JNEUROSCI.1691-21.2021 PMC8802923

[60] Staurengo-Ferrari L, Green PG, Araldi D, Ferrari LF, Miaskowski C, Levine JD (2021) Sexual dimorphism in the contribution of neuroendocrine stress axes to oxaliplatin-induced painful peripheral neuropathy. Pain 162(3):907–918. 10.1097/j.pain.0000000000002073 32947545 10.1097/j.pain.0000000000002073 PMC7886966

[61] Rakshith HT, Lohita S, Rebello AP, Goudanavar PS, Raghavendra Naveen N (2023) Sex differences in drug effects and/or toxicity in oncology. Curr Res Pharmacol Drug Discov 4:100152. 10.1016/j.crphar.2022.100152 36714036 10.1016/j.crphar.2022.100152 PMC9881040

[62] Mogil JS, Bailey AL (2010) Sex and gender differences in pain and analgesia. Prog Brain Res 186:141–57. 10.1016/B978-0-444-53630-3.00009-9 21094890 10.1016/B978-0-444-53630-3.00009-9

[63] Rades D, Bartscht T, Rody A, Yu NY, Ballegaard M (2025) Chemotherapy-induced moderate to severe peripheral neuropathy in patients receiving adjuvant radiotherapy for breast cancer. Anticancer Res 45(5):2123–2135. 10.21873/anticanres.17587 40295075 10.21873/anticanres.17587

[64] Cohen J (1988) Statistical power analysis for the behavioral sciences, 2 nd ed. Lawrence Erlbaum Associates, Hillsdale, NJ

[65] Guyatt GH, Osoba D, Wu AW, Wyrwich KW, Norman GR, Clinical Significance Consensus Meeting G (2002) Methods to explain the clinical significance of health status measures. Mayo Clin Proc 77(4):371–83. 10.4065/77.4.371 11936935 10.4065/77.4.371

[66] Osoba D, Rodrigues G, Myles J, Zee B, Pater J (1998) Interpreting the significance of changes in health-related quality-of-life scores. J Clin Oncol 16(1):139–144 9440735 10.1200/JCO.1998.16.1.139

[67] Tofthagen CS, Cheville AL, Loprinzi CL (2020) The physical consequences of chemotherapy-induced peripheral neuropathy. Curr Oncol Rep 22(5):50. 10.1007/s11912-020-00903-0 32323068 10.1007/s11912-020-00903-0

[68] Li L, Zhang S, Dobson J (2019) The contribution of small and large sensory afferents to postural control in patients with peripheral neuropathy. J Sport Health Sci 8(3):218–227. 10.1016/j.jshs.2018.09.010 31193300 10.1016/j.jshs.2018.09.010 PMC6523875

[69] Trendowski MR, Lusk CM, Ruterbusch JJ, Seaton R, Simon MS, Greenwald MK et al (2021) Chemotherapy-induced peripheral neuropathy in African American cancer survivors: risk factors and quality of life outcomes. Cancer Med 10(22):8151–61. 10.1002/cam4.4328 34687150 10.1002/cam4.4328 PMC8607253

